# Effect of Rearing in Small-Cell Combs on Activities of Catalase and Superoxide Dismutase and Total Antioxidant Capacity in the Hemolymph of *Apis mellifera* Workers

**DOI:** 10.3390/antiox12030709

**Published:** 2023-03-13

**Authors:** Piotr Dziechciarz, Aneta Strachecka, Grzegorz Borsuk, Krzysztof Olszewski

**Affiliations:** 1 Subdepartment of Apidology, Institute of Biological Basis of Animal Production, Faculty of Animal Sciences and Bioeconomy, University of Life Sciences in Lublin, 20-950 Lublin, Poland; 2 Department of Invertebrate Ecophysiology and Experimental Biology, University of Life Sciences in Lublin, 20-950 Lublin, Poland

**Keywords:** small-cell combs, *Apis mellifera*, hemolymph, catalase, superoxide dismutase, total antioxidant capacity

## Abstract

Honeybee nests constructed without man-made wax foundation have significantly more variability of cell widths/sizes than those in commercially-kept colonies. The effects of this natural variability in comb cell widths on individual and colony traits have not been explained to date. The investigation of this problem can lead to new findings about the biology, physiology, and possibly, the evolution of the honeybee. The aim of the study was to compare the catalase and superoxide dismutase activities and the total antioxidant capacity levels in the hemolymph of honeybee workers reared in small-cell combs and standard-cell combs in colonies kept simultaneously on standard- and small-cell combs. The ratio of the small-cell combs to the standard-cell combs in the nest was 1:1. The workers reared in small-cell combs were characterized by higher antioxidant activities in the hemolymph than those reared in standard-cell combs. Consequently, their hemolymph had a greater antioxidant capacity, which indicates that they may be better predisposed to be foragers than workers reared in standard-cell combs. To describe the physiological differences between worker bees reared in small- and standard-cell combs in the same colony, the role of the considerable variation in the cell width in natural combs built without the use of artificially produced wax foundation is worth elucidating. The comparison of the apiary and cage experiments indicated that changes in antioxidant activities predominantly result from worker activities, especially those requiring the intensification of metabolism, rather than the age of the worker bees. To reduce the impact on the results of random environmental factors potentially present in one-season studies of honeybee research, investigations should preferably be carried out over a few consecutive years.

## 1. Introduction

An adequate nutrient supply and resistance to the pressure posed by parasites, predators, and other adverse environmental factors are the main determinants of survival for all animals, including the superorganism constituted by a colony of social insects [1]. The honeybee is a social insect, and the main problems in contemporary apiculture are caused by external environmental stressors and internal nest factors that affect both individual bees and the colony as a whole [2]. The greatest risk of exposure to stressors in the external environment is faced by the forager caste during flights for food and water. Contaminants encountered by these bees in the environment around the bee colony are transported on their bodies and enter the colony along with food and water, thus affecting the other bees and the brood in the nest [3,4].

The maintenance of homeostasis in individual honeybees and, consequently, in entire colonies, is ensured by, e.g., the mitigation of the impact of negative factors by the immune and antioxidant systems. The effectiveness of the bee immune system is associated with the degradation of its protein structures and can be impaired by the oxidative stress that results from an imbalance between the generation of reactive oxygen species (ROS) and the antioxidant capacity of the organism [5,6,7]. ROS cause the oxidation of proteins, RNA, and DNA, as well as the peroxidation of membrane lipids. These destructive reactions accelerate the aging processes and induce carcinogenesis and cell death [8]. Despite the small number of genes encoding antioxidant proteins, in comparison with such insects as *Drosophila melanogaster* or *Anopheles gambiae* [9], honeybees have effective mechanisms that primarily involve superoxide dismutase (SOD) and catalase (CAT) in the hemolymph to protect their organisms against the harmful effects of ROS [9,10]. The importance of the insect antioxidant system is associated with their quick metabolism, which naturally generates large amounts of free radicals [11].

SOD (superoxide dismutase) converts O_2−_ to hydrogen peroxide (H_2_O_2_), which is then diffused in the cell and removed by, e.g., CAT (catalase). CAT is one of the most important and effective antioxidant enzymes. It is involved in the decomposition of hydrogen peroxide into water (H_2_O) and molecular oxygen (O_2_) [12,13,14].

The interaction between different antioxidants provides better protection against the effects of ROS than could any single compound. The oxidative changes and efficiency of the entire antioxidant system in bees are quantified in the total antioxidant capacity (TAC) analysis [15,16,17]. Intracellular TAC is mainly attributed to the enzyme system, whereas plasma TAC is mainly related to dietary low molecular weight antioxidants. These compounds are quickly utilized during ROS scavenging; hence, they must be replenished or replaced with new compounds contained in food. Plasma TAC is modulated in two ways: either by oxidative free radicals or by dietary compounds with antioxidant properties. Therefore, TAC can be regarded as indicating the homeostasis between ROS and antioxidants (identified and unidentified, measurable and non-measurable) more efficiently than does the concentration of single antioxidants [18].

To date, it has been confirmed that the effectiveness of the antioxidant system in honeybee workers is influenced by such external environmental factors as pesticides [19,20,21], acaricides used against Varroa destructor [22], nutritional supplements [23,24,25,26], and the electromagnetic field [27].

Combs are inanimate components of the honeybee nest. They are built by workers from wax produced in wax glands. They are used for offspring rearing, food storage, and information exchange between colony members through dancing and pheromones [28]. They are also referred to as the “skeleton of the bee colony”. Given the variety of its functions, Tautz [28] defines the comb as the most important organ of the bee colony superorganism. Currently, honeybee colonies kept in developed countries build their combs on thin sheets of beeswax bearing imprinted cell outlines and fitted in wooden frames. These wax foundations are produced artificially, and their use eliminates any variability in the width/size of cells in colony worker combs. In contrast, the nests of colonies with natural combs built without the wax foundation exhibit substantial variability in the cell width in worker and drone combs [29]. In a study conducted by Maggi et al. [29], worker brood was reared in 4.17–6.75-mm-wide cells and drone brood cells were 5.04–8.05 mm wide.

Our team was the first to attempt to explain the role of such considerable variation in the cell width in a bee colony. As shown by our research, female workers reared in the so-called small cells (approx. 4.90 mm wide) differ from those reared in standard cells (approx. 5.50 mm wide) in both morphological [30] and physiological [31] traits. We were the first to place two types of combs (with small and standard cells) in a bee colony nest to mimic natural nests built without the wax foundation [30]. Based on the comparison of the morphological traits and the activities of the proteolytic system in the hemolymph of the workers reared in small- and standard-cell combs, we hypothesized that the workers reared in small cells were predisposed to serve as foragers, whereas those from standard cells performed various tasks within the nest, e.g., nursing [31]. Additionally, we found a higher rate of dead brood removal from the small-cell rather than standard-cell combs [32], which indicates a possibility of modifying the behavior of worker bees by choosing the width of comb cells.

The aim of the study was to determine the effect of the width of comb cells (small vs. standard) on the activities of the antioxidant system in worker bees reared in combs with different cell widths.

## 2. Materials and Methods

The experiment was conducted at the University of Life Sciences in Lublin (Poland). The apiary experiments (location of the apiary: 51.224039 N–22.634649 E) and the laboratory cage tests were conducted each year; the same experimental design was repeated in three consecutive years: 2020, 2021, and 2022. This was to avoid the impact of random environmental factors that may appear in one-season analyses.

### 2.1. Acquisition of Bees

Five foster colonies of workers were used in the apiary and laboratory experiments each year. The colonies exhibited similar strength and structure. The workers occupied the same number of combs, and the areas of capped and uncapped brood cell surfaces were quite similar. The queens in the foster colonies were naturally inseminated sisters of the same age. Buckfast bee colonies were used due to their good adaptation to living on small-cell combs [30,31,32]. All the colonies were kept in Dadant Blatt hives, and their nests contained two types of combs: small-celled (cell width approx. 4.90 mm) and standard-celled (cell width approx. 5.50 mm) [31]. The design of the small- and standard-cell combs in the brood chamber was consistent with that presented by Dziechciarz et al. [32]. The worker bees were reared in the small-cell (SMC) and standard-cell (STC) combs. At the age of 1 day, some worker bees were labeled [32]. The labeled workers were used in the apiary experiment, whereas the unlabeled individuals were used in the laboratory experiment.

### 2.2. Apiary Experiment

Each year, 1-day-old labeled workers from each foster colony and each comb type (small/standard cells) were placed in five colonies kept in six-comb hives. In total, there were 1500 SMC workers (reared in small-cell combs) and 1500 STM workers (reared in standard-cell combs) [32]. The colonies had similar strength and structure. The workers occupied the same number of combs, and the areas of capped and uncapped brood cell surfaces were quite similar. Each colony had a normally ovipositing queen, five combs with different-age brood, and one comb with honey and bee brood. The queens in these colonies were sisters of the same age. The workers reared in each of the foster colonies were placed in a separate colony [32]. In the apiary experiment, we used colonies kept on six combs, as it was easier to collect the labeled workers for hemolymph sampling. During this experiment, moderate nectar flow, mainly from *Tilia* spp., was noted.

### 2.3. Laboratory Experiment

Each year, workers from each foster colony and each type of comb (small/standard cells), were placed in three cages. Two groups of cages were formed in this way, one group with the SMC workers and the other with the STC workers. Every year, each group consisted of 15 cages (5 colonies with 1 SMC comb and 1 STC comb in each × 3 cages of each type of comb = 15 cages with SMC workers and 15 cages with STC workers). The cages containing the worker bees were kept in a room at a constant temperature of 25 °C. Throughout the experiment, the worker bees were fed *ad libitum* with syrup composed of water and sucrose at a 1:1 ratio. To avoid fermentation, the syrup was replaced with a fresh one every day.

### 2.4. Hemolymph Collection

On days 7, 14, and 21, hemolymph was collected from the SMC and STC workers kept in the colonies and cages, with the exception of 2020, when no hemolymph was sampled from 21-day-old worker bees, as the bees in all cages had died before that day. The hemolymph was collected at the site of antenna detachment [33]. The SMC and STC workers were chosen randomly from each of the five colonies kept on the six combs. Hemolymph collected from five worker bees constituted one sample [31]. In the laboratory experiment, one hemolymph sample, consisting of the hemolymph collected from five randomly selected worker bees (SMC/STC), was obtained from each cage and each group of workers. Hemolymph was also sampled from 1-day-old workers from each foster colony and each comb type.

The hemolymph collected from each group of five worker bees was transferred into a separate Eppendorf tube (0.5 mL) filled with 150 µL of 0.6% NaCl and placed in a CoolBox to prevent melanization [25,31,34]. The number of hemolymph samples collected in consecutive years is shown in Table 1. To eliminate the impact on the results from potential *Nosema* spp. fungal infection of the workers, each group of five worker bees from which hemolymph was sampled in the apiary and laboratory experiments was tested for the presence of *Nosema* spp. spores [35]. The level of infection was assessed in 7-, 14-, and 21-day-old workers.

### 2.5. Determination of Antioxidant Activities

We decided to analyze hemolymph in the present study, as its parameters are a good source of information about the physiological status of the organism [36]. The antioxidant activities were assessed with the spectrophotometric method using commercial kits. Catalase enzyme (CAT) activity in the worker hemolymph was determined using the Sigma-Aldrich kit (CAT-100). One unit of CAT is the amount of the enzyme that decomposes 1 µmol of H_2_O_2_ per minute at 25 °C. The H_2_O_2_ decomposition was measured at a wavelength of 520 nm and the results were expressed as U/mL of the catalase enzyme in the sample volume. The activity of superoxide dismutase (SOD) was determined using the Sigma-Aldrich 19160 SOD determination kit, with one unit inhibiting the rate of reduction of cytochrome c by 50% in a coupled system using xanthine and xanthine oxidase in the sample volume. The total antioxidant capacity (TAC) level in the hemolymph of the worker bees was assessed using a Cell Biolabs STA-360 kit. The antioxidant capacity of unknown samples was expressed as μM of copper reducing equivalents and was proportional to the sample’s total antioxidant capacity in the sample volume. The samples and sample volumes were prepared according to the aforementioned enzyme analysis protocols.

### 2.6. Measurements of Comb Cell Width

Each small- and standard-cell comb where the workers were reared for the apiary and laboratory experiments was photographed in the center of each comb half on one side of the comb. Next, in each half, the widths of the 10 adjacent cells making contact with the vertical side walls were measured following the procedure used by [30]. Each year (2020, 2021 and 2022), 100 cells (5 combs × 2 measurements of 10 cells per combs) were measured in each type of the comb (i.e., small- and standard-cell comb), respectively [31].

### 2.7. Statistical Analysis

The statistical analysis of the results was performed using Statistica software formulas, version 13.3 (2017) for Windows, StatSoft Inc., Tulsa, OK, USA.

In the apiary and laboratory experiments, the data distribution in the assessment of the impact of the year on the CAT and SOD activities and TAC levels was analyzed separately for the SMC and STC workers using the Kolmogorov–Smirnoff test. In the analysis of the effect of the year on the CAT and SOD activities and TAC levels separately for SMC and STC workers, ANOVA was used for data with a normal distribution and the Kruskal–Wallis test was used for data with a non-normal distribution.

In the apiary and laboratory experiments, the data distribution in the assessment of the impact of the age on the activities of CAT, SOD, and TAC in each of the three years (2020, 2021, 2022) was analyzed separately for the SMC and STC workers with the Shapiro–Wilk test. The effect of the age on the CAT and SOD activities and TAC levels assessed separately for the SMC and STC workers was analyzed with the use of ANOVA (data with a normal distribution) and the Kruskal–Wallis test (non-normally distributed data).

The CAT, SOD, and TAC results in the 1-day-old SMC and STC workers were compared with the use of Student’s t-test for dependent samples (normally distributed data) and the pairwise Wilcoxon test (non-normally distributed data). The distribution of these data was analyzed using the Shapiro–Wilk test.

In the apiary experiment, the data on the activities of CAT and SOD and levels of TAC within the age groups (7 d, 14 d, and 21 d) were compared between SMC and STC with Student’s t-test for dependent samples (normal distribution) and with the pairwise Wilcoxon test (non-normal distribution). The distribution of these data was analyzed using the Shapiro–Wilk test. In turn, in the laboratory experiment, the levels of CAT, SOD and TAC within the age groups (7 d, 14 d, and 21 d) were compared between SMC and STC with the Student’s t-test for independent samples (normal distribution) and with the Mann–Whitney U test (non-normal data distribution). The distribution of these data was analyzed with the use of the Shapiro–Wilk test.

The relationship between the width of the comb cells in the foster colonies and the year (2020, 2021, 2022) was assessed separately for the small-cell combs (*n* = 300) and the standard-cell combs (*n* = 300) using the Kruskal–Wallis test. The distribution of these data was analyzed with the Kolmogorov–Smirnoff test. The width of the small-cell combs in the foster colonies (*n* = 300) was compared with the width of the standard-cell combs (*n* = 300) collectively for the three years using the Mann–Whitney U test, as the effect of the year was not significant for the width in either the small- or standard-cell combs. The distribution of these data was analyzed with the Kolmogorov–Smirnoff test.

## 3. Results

### 3.1. Comb Cell Width

The cell width in the small-cell combs and in the standard-cell combs in the foster colonies did not significantly differ statistically between the years (respectively: H = 2.933, df = 2, *p* = 0.229, *n* = 300; H = 4.407, df = 2, *p* = 0,111, *n* = 300; Kruskal–Wallis test).

The width of the small cells was significantly smaller (*p* ≤ 0.01; *n* = 300; Mann–Whitney U test) than that of the standard cells. The mean value of the width of small cells in the combs of the foster colonies was 4.96 mm (SD = 0.043), whereas the width of standard cells in the combs of the foster colonies was 5.57 mm (SD = 0.054).

### 3.2. Infection of Worker Bees by Nosema spp.

Neither of the bee samples for which hemolymph was collected in the apiary and laboratory experiments contained *Nosema* spp. spores. This allows the analysis to eliminate from the findings the effects of infestation with this parasite.

### 3.3. SOD and CAT Activities and TAC Levels

In both the SMC and STC workers, the year (i.e., 2020, 2021, and 2022) had a significant effect on the SOD and CAT activities and TAC levels in the hemolymph (Table 2). In all years, the age (1 d, 7 d, 14 d, and 21 d) exerted a significant effect on the SOD and CAT activities and the TAC levels in the hemolymph of both the SMC and STC workers (Table 2). The SOD activity in the STC workers in 2020 was an exception.

**Figure 1:**
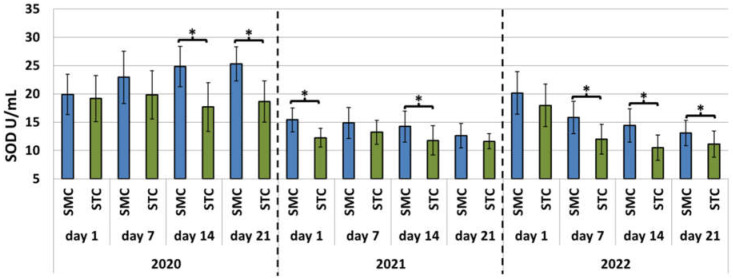
Apiary experiment—Superoxide dismutase (SOD) activities in the hemolymph of workers in three consecutive years. SMC—workers reared in small-cell combs; STC—workers reared in standard-cell combs; *—differences between the SMC and STC workers within the age group are significant at *p* ≤ 0.05; vertical bars indicate standard deviation.

**Figure 2:**
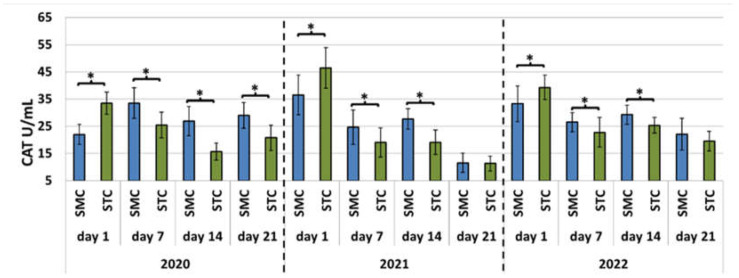
Apiary experiment—Catalase (CAT) activities in the hemolymph of workers in three consecutive years. SMC—workers reared in small-cell combs; STC—workers reared in standard-cell combs; *—differences between the SMC and STC workers within the age group are significant at *p* ≤ 0.05; vertical bars indicate standard deviation.

**Figure 3:**
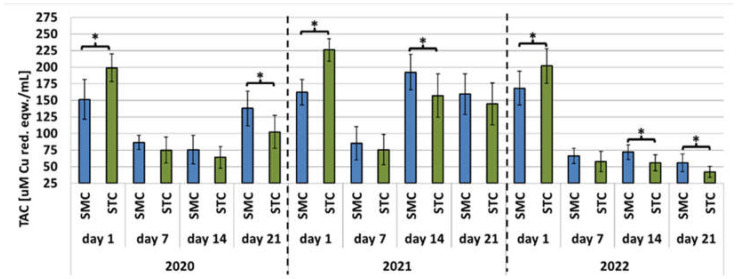
Apiary experiment—Total antioxidant capacity (TAC) levels in the hemolymph of workers in three consecutive years. SMC—workers reared in small-cell combs; STC—workers reared in standard-cell combs; *—differences between the SMC and STC workers within the age group are significant at *p* ≤ 0.05; vertical bars indicate standard deviation.

**Figure 4:**
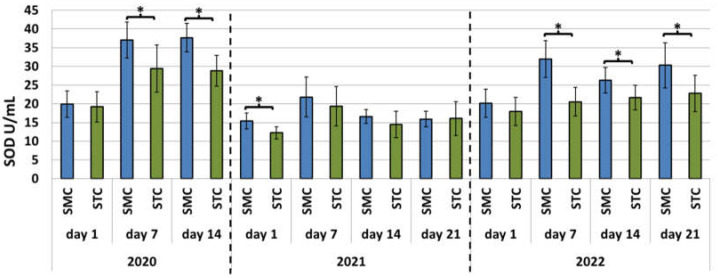
Laboratory experiment—Superoxide dismutase (SOD) activities in the hemolymph of workers in three consecutive years. SMC—workers reared in small-cell combs; STC—workers reared in standard-cell combs; *—differences between the SMC and STC workers within the age group are significant at *p* ≤ 0.05; vertical bars indicate standard deviation.

**Figure 5:**
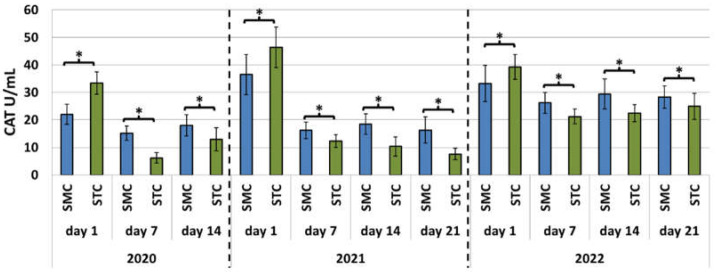
Laboratory experiment—Catalase (CAT) activities in the hemolymph of workers in three consecutive years. SMC—workers reared in small-cell combs; STC—workers reared in standard-cell combs; *—differences between the SMC and STC workers within the age group are significant at *p* ≤ 0.05; vertical bars indicate standard deviation.

**Figure 6:**
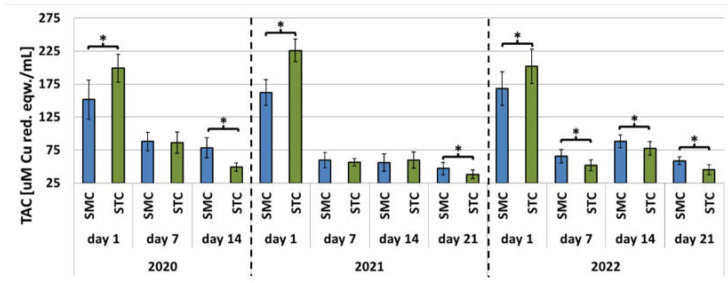
Laboratory experiment—Total antioxidant capacity (TAC) levels in the hemolymph of workers in three consecutive years. SMC—workers reared in small-cell combs; STC—workers reared in standard-cell combs; *—differences between SMC and STC workers within the age group are significant at *p* ≤ 0.05; vertical bars indicate standard deviation.

In each year of the apiary experiment, the SOD and CAT activities and TAC levels were higher in the 7-, 14-, and 21-day-old SMC workers than in the STC workers (Figure 1, Figure 2 and Figure 3, Table 3). The highest number of statistically significant differences was noted in the case of the SOD and CAT activities. Opposite results were obtained in the analysis of the CAT activities and TAC levels in the 1-day-old workers, i.e., statistically significantly lower values of these parameters were recorded in the group of the SMC workers vs. the STC workers (Figure 1, Figure 2 and Figure 3, Table 3). In turn, the SOD activities in the 1-day-old workers were higher in the STC group, but the differences were statistically significant only in 2021 (Figure 1, Figure 2 and Figure 3, Table 3). In the laboratory experiment, the trends observed in the groups of the 7-, 14-, and 21-day-old workers were very similar to those in the apiary experiment (Figure 4, Figure 5 and Figure 6, Table 3).

Different trends were evident in the SOD activities in the apiary and laboratory experiments each year (Figure 1 and Figure 4). In the SMC workers in the apiary experiment conducted in 2020, the 1-day-old workers exhibited the lowest activities of the enzymes, but they increased with age (Figure 1). Similar values of the SOD activities were recorded for the STC workers. In the group of the SMC workers in 2021, the highest SOD activities were determined for the hemolymph of the 1- and 7-day-old workers, but the activities declined in the older workers. In turn, the activity persisted at a similar level in the group of the STC workers. In 2022, the highest SOD activities were noted in the 1-day-old SMC and STC workers, but their values decreased with age. In the laboratory experiment conducted in 2020 and 2022, the lowest SOD activities were recorded in the samples collected from the 1-day-old SMC and STC workers. The SOD activities in the hemolymph of the older workers were higher and persisted at a similar level (Figure 4). In 2021, the lowest SOD activities were noted in the groups of the 1-day-old SMC and STC workers. The highest activity was recorded for the 7-day-old workers, but the activities in the hemolymph of the older workers decreased.

With the exception of the apiary experiment conducted in 2020, in all of the apiary and laboratory experiments, the CAT activities were higher in the 1-day-old workers than in the older workers (Figure 2 and Figure 5). In turn, in the apiary experiment, the values of the enzyme declined in the older SMC and STC workers after the 14th day of life in 2021 and 2022 and increased in 2020 (Figure 2). In the laboratory experiment, the activities of the enzyme persisted at the same level in both the SMC and STC workers (Figure 5).

In the laboratory experiment, the trends in the TAC parameter were very similar in the SMC and STC workers in each year. The highest TAC levels were recorded in the 1-day-old workers; next, they decreased significantly and remained at similar levels in the older workers (Figure 6). In the apiary experiments conducted in 2020 and 2022, the SMC and STC workers exhibited similar trends in TAC levels (Figure 3). The highest values were recorded in the 1-day-old workers; they declined or remained at similar levels in the older workers. The 21-day-old workers in 2020 were an exception, as the TAC levels increased in comparison with the 1- and 7-day-old workers. In the STC group analyzed in 2021, the highest TAC values were found in the 1-day-old workers. Their levels decreased significantly in the samples collected from the 7-day-old workers, increased significantly in the hemolymph of the 14-day-old insects, and declined again in the group of the 21-day-old insects. The TAC levels in the 7-day-old STC workers also decreased significantly in comparison with the 1-day-old workers; on day 14, the levels increased and exceeded the values recorded for the 1-day-old group. Next, the TAC levels decreased in the 21-day-old group, as they did in the SMC workers.

## 4. Discussion

### 4.1. SOD and CAT Activities and TAC Levels in the SMC and STC Workers

The present study showed that the CAT and SOD activities and TAC levels on days 7, 14, and 21 were usually higher in the hemolymph of the SMC workers in comparison with the STC group. The same trends were observed both in the apiary experiment and in the laboratory cage tests. With the exception of the SOD activities, opposite results were obtained in the group of 1-day-old insects in comparison with the 7-, 14-, and 21-day-old worker bees. Thus, we demonstrated a significant effect of the width of worker comb cells on the activities of antioxidants in worker bee hemolymph. We described very similar trends in our previous study, showing a significant effect of different widths of worker comb cells (small vs. standard) on the total protein content and the activities of proteolytic enzymes and their inhibitors in the hemolymph [31]. As in the case of the activities of antioxidants on days 7, 14, and 21, the activities of proteolytic enzymes and their inhibitors in the hemolymph were higher in the worker bees reared in small-cell combs than in standard-cell combs. In contrast, the total protein content in the hemolymph was lower. Opposite results were obtained in the group of 1-day-old workers in comparison with the 7-, 14-, and 21-day-old bees.

Higher CAT and SOD activities and TAC levels determined in the 7-, 14-, and 21-day-old SMC workers in the apiary and laboratory conditions contributed to higher ROS scavenging capacity in comparison with the STC workers. Interestingly, with the exception of SOD activity, opposite results were obtained in the group of the 1-day-old workers, as in the case of the previously reported activities of proteases and their inhibitors [31]. Elevated activities of antioxidant enzymes in worker bees and thus the ability to neutralize free radicals through an increased expression of antioxidant genes may result from the quality of nutrition, including the protein content in the larval diet [37,38]. The samples collected from the 1-day-old SMC workers exhibited higher protein content than those from the STC workers. The protein level declined with age, as it may have been utilized for, e.g., the synthesis of proteolytic enzymes and their inhibitors [31].

### 4.2. Age-Related Changes in SOD and CAT Activities and TAC Levels in the Workers

In many organisms [39,40,41], including bees [13,42], the ability to stimulate the activities of heat shock proteins and antioxidants decreases with age. Different results were obtained by Paleolog et al. [21] and Skowronek et al. [34] in apiary experiments and by Skowronek et al. [25] in laboratory analyses; they demonstrated an age-related increase in the activity of the antioxidant system in worker bees. The findings obtained in the apiary experiments may reflect the adaptation of the worker bees to function as foragers outside the nest in an environment exposed to pollution [43,44,45]. Furthermore, the increased activities of detoxification enzymes in older worker bees seem to indicate their biochemical adaptation to the forager work, compensating for the age-related decline in protein content [43]. However, in the case of pesticide poisoning, e.g., with imidacloprid, the activity of the antioxidant system in workers exposed to the pesticide was found to decline with age (20 days old) in comparison with imidacloprid-untreated 1-day-old workers [21]. In contrast, the activity of the antioxidant system in laboratory experiments was reported to increase with age [25].

However, a direct comparison of the present results with the findings reported by Paleolog et al. [21] and Skowronek et al. [25] is difficult, as the authors calculated the antioxidant activity per protein mass unit without specifying the protein content per solution volume unit in the worker age groups. This method of data presentation does not provide explicit information about whether the significant increase in the antioxidant activity is related to an actual increase in the activities of the antioxidants or to the significant decrease in the protein content without a significant change in the antioxidant activities. Such a relationship was indicated in other studies that compared changes in protein concentrations in the hemolymph solution with changes in the activities of proteases [23,24,31]. In the present study, to exclude the impact of fluctuations in protein concentrations on antioxidant activities, we calculated the SOD, CAT and TAC values per solution volume unit, without converting the result into a protein mass unit.

In the apiary experiment conducted in the present study, the antioxidant activities tended to decrease with age in both the SMC and STC worker groups. The greatest decrease was recorded between days 1 and 7. In the cage experiment, with the exception of SOD, the activity of antioxidants decreased significantly between days 1 and 7 (as in the apiary experiment), but then persisted at a similar level. The decrease in the antioxidant activities between days 1 and 7 may be associated with the change in the habitat from the conditions prevailing in the capped comb cell with a constant high temperature (34.44–35.39 °C) [46] to the less precisely regulated conditions of the nest and external environment. We collected hemolymph from 1-day-old worker bees immediately after their emergence from the comb cells; therefore, their metabolism may not yet have reacted to the change in the conditions. In turn, the significant decrease in the CAT activities and TAC levels between days 1 and 7 may have been a result of the decrease in the ambient temperature. In contrast, the gradual decrease in SOD and CAT activity in the older workers (between days 7 and 21) was probably caused by the age-related decline in the ability to stimulate antioxidant activity induced by prolonged exercise [13]. Similarly, Williams et al. [47] found that the ability of the antioxidant system to deactivate ROS decreased with age in nurses and foragers up to the loss of the ability in aged honeybees (30–32 days). This resulted in reduced productivity and a shorter lifespan [47]. Our assumption was confirmed by the laboratory experiment results, which showed that the CAT activities and TAC levels declined between days 1 and 7 in the SMC and STC workers and then persisted at a similar level between days 7 and 21, probably because these bees did not take up any activities in accordance with their role in the colony. These observations indicate that changes in the antioxidant activity are largely a consequence of the activities performed by worker bees, especially those requiring an intensification of metabolism, rather than their age.

The trends in SOD activity were opposite to those in CAT activities in both the apiary and laboratory experiments. No decline in SOD activity was observed between days 1 and 7. In the laboratory experiment, the SOD activities increased and persisted at a similar level between days 7 and 21. No significant changes in this parameter in caged workers between days 9 and 26 were found by [13]. In turn, the activities of SOD in our apiary experiment were dependent on the year. In 2020, they increased with age in the SMC worker group and persisted at a similar level in the samples collected from the STC workers. In 2021 and 2022, the activities decreased with age in both the SMC and STC workers, and the decline noted in 2021 was substantially lower than in 2022. Different trends were observed in the age-related CAT and TAC changes in the bees in the apiary experiment in the particular years of the study. In the laboratory experiment, the trends in the levels of all the antioxidants were similar. This indicates a significant impact of environmental factors on the results of our apiary experiments; therefore, to reduce the risk of an effect of random environmental factors on the results of one-season investigations of honeybees, it is advisable to continue the research over a few consecutive years.

## 5. Conclusions

Workers reared in small-cell combs exhibit higher hemolymph antioxidant activities than those from standard-cell combs. Consequently, their hemolymph has a greater antioxidant capacity.

In terms of physiological differences between worker bees reared in small- and standard-cell combs in the same bee colony, it is necessary to elucidate the role of the considerable variation in the cell width in natural combs built without the artificial wax foundation.

To reduce the effect of random environmental factors that may be encountered in one-season studies on the results of research on honeybees, it is advisable to continue such investigations over several consecutive years.

## Figures and Tables

**Table 1:** Number of hemolymph samples in each age group of the workers (1 d, 7 d, 14 d, and 21 d) in the apiary and laboratory experiments in the three consecutive years.

Age	Group	Year					
		2020		2021		2022	
		Apiary	Laboratory	Apiary	Laboratory	Apiary	Laboratory
1 day	SMC	20	20	10	10	10	10
	STC	20	20	10	10	10	10
7 days	SMC	20	15	20	15	20	15
	STC	20	15	20	15	20	15
14 days	SMC	20	15	20	15	20	15
	STC	20	15	20	15	20	15
21 days	SMC	10	-	20	15	20	15
	STC	10	-	20	15	20	15

SMC—workers reared in small-cell combs; STC—workers reared in standard-cell combs.

**Table 2:** Effect of the year (i.e., 2020, 2021, and 2022) and age (1 d, 7 d, 14 d, and 21 d) on the hemolymph parameters of workers reared in small- and standard-cell combs in the apiary and laboratory experiments.

Hemolymph Parameters	Impact of the Year		Impact of the Age					
			2020		2021		2022	
	SMC	STC	SMC	STC	SMC	STC	SMC	STC
SOD **Apiary**	H = 118.34	H = 79.76	F = 14.06	F = 16.92	H = 28.26	F = 3.92	H = 50.16	H = 38.83
	df = 2	df = 2	df = 3	df = 3	df = 3	df = 3	df = 3	df = 3
	*p* = 0.00	*p* = 0.00	*p* = 0.00	*p* = 0.58	*p* = 0.00	*p* = 0.00	*p* = 0.00	*p* = 0.00
CAT **Apiary**	F = 7.58	H = 26.13	H = 17.73	F = 30.75	F = 46.76	H = 49.87	F = 11.84	H = 53.46
	df = 2	df = 2	df = 3	df = 3	df = 3	df = 3	df = 3	df = 3
	*p* = 0.00	*p* = 0.00	*p* = 0.00	*p* = 0.00	*p* = 0.00	*p* = 0.00	*p* = 0.00	*p* = 0.00
TAC **Apiary**	H = 49.00	H = 37.73	H = 54.48	H = 45.96	H = 108.48	H = 61.72	H = 64.90	H = 67.18
	df = 2	df = 2	df = 3	df = 3	df = 3	df = 3	df = 3	df = 3
	*p* = 0.00	*p* = 0.00	*p* = 0.00	*p* = 0.00	*p* = 0.00	*p* = 0.00	*p* = 0.00	*p* = 0.00
SOD **Laboratory**	H = 65.80	H = 53.07	H = 21.84	F = 13.07	H = 16.48	H = 14.04	H = 38.88	F = 4.56
	df = 2	df = 2	df = 2	df = 2	df = 3	df = 3	df = 3	df = 3
	*p* = 0.00	*p* = 0.00	*p* = 0.00	*p* = 0.00	*p* = 0.00	*p* = 0.00	*p* = 0.00	*p* = 0.01
CAT **Laboratory**	F = 44.15	H = 47.61	F = 11.20	H = 30.85	H = 27.05	H = 34.33	F = 5.30	H = 42.54
	df = 2	df = 2	df = 2	df = 2	df = 3	df = 3	df = 3	df = 3
	*p* = 0.00	*p* = 0.00	*p* = 0.00	*p* = 0.00	*p* = 0.00	*p* = 0.00	*p* = 0.00	*p* = 0.00
TAC **Laboratory**	H = 27.29	H = 6.24	H = =20.54	H = 34.31	H = 30.25	H = 40.91	H = 54.46	H = 55.40
	df = 2	df = 2	df = 2	df = 2	df = 3	df = 3	df = 3	df = 3
	*p* = 0.00	*p* = 0.04	*p* = 0.00	*p* = 0.00	*p* = 0.00	*p* = 0.00	*p* = 0.00	*p* = 0.00

SMC—workers reared in small-cell combs; STC—workers reared in standard-cell combs, H—value of statistics for the Kruskal–Wallis test; F–value of Fisher’s test for ANOVA; df—number of degrees of freedom; *p*—probability value.

**Table 3:** Changes in antioxidant activities in workers reared in small-cell combs (SMC) in comparison with workers reared in standard-cell combs (STC) at the ages of 1, 7, 14, and 21 days in the apiary and laboratory experiments.

Trait	2020				2021				2022			
	1 d	7 d	14 d	21 d	1 d	7 d	14 d	21 d	1 d	7 d	14 d	21 d
SOD **Apiary**	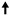 n.s.	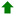	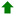	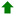	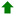	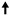 n.s.	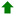	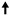 n.s.	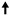 n.s.	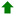	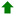	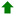
CAT **Apiary**	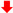	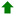	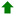	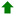	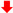	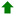	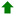	**=** n.s.	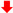	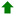	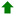	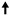 n.s.
TAC **Apiary**	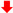	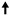 n.s.	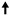 n.s.	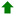	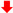	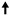 n.s.	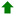	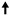 n.s.	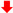	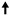 n.s.	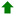	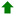
SOD **Laboratory**	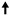 n.s.	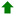	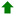	**-**	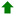	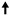 n.s.	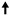 n.s.	**=** n.s.	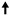 n.s.	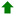	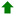	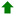
CAT **Laboratory**	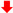	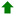	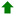	**-**	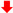	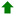	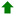	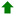	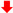	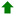	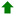	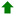
TAC **Laboratory**	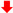	**=** n.s.	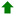	**-**	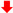	**=** n.s.	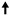 n.s.	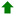	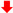	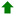	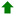	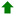

CAT—Catalase enzyme activity; SOD—Superoxide dismutase enzyme activity; TAC—Total antioxidant capacity; 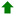
—green arrow indicates that the value of the trait is statistically significantly higher in the SMC workers than the STC workers; 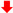
—red arrow indicates that the value of the trait is statistically significantly lower in the SMC workers than the STC workers; n.s.—statistically insignificant difference between the SMC and STC workers; 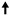
—black arrow indicates that the value of the trait is higher in the SMC workers than the STC workers but without statistical significance; **=**—equality sign indicates that the value of the trait in the SMC workers is close or equal to the trait value in the STC workers.

## Data Availability

The data presented in this study are available on request from the corresponding author.
